# Isolated Anti-HBc: Significance and Management

**DOI:** 10.3390/jcm9010202

**Published:** 2020-01-11

**Authors:** Florian MORETTO, François-Xavier CATHERINE, Clémentine ESTEVE, Mathieu BLOT, Lionel PIROTH

**Affiliations:** 1 Infectious Diseases Department, Dijon University Hospital, 21079 Dijon, France; florian.moretto@chu-dijon.fr (F.M.); francois-xavier.catherine@chu-dijon.fr (F.-X.C.); clementine.esteve@chu-dijon.fr (C.E.); mathieu.blot@chu-dijon.fr (M.B.); 2 INSERM CIC 1432, Module Plurithématique, University of Burgundy, 21079 Dijon, France

**Keywords:** viral hepatitis, isolated anti-HBc antibodies, immunosuppression, hepatitis B infection, HBV reactivation, management

## Abstract

Hepatitis B virus (HBV) infection is prevalent worldwide and is associated with dramatic levels of morbidity and mortality. Isolated anti-HBc (IAHBc) is a particular serological pattern that is commonly found in immunocompromised patients. There is ongoing debate regarding the management of patients with IAHBc. Herein, we summarize the current guidelines and the newest evidence. The frequency of IAHBc is variable, with a higher prevalence in some populations, such as persons living with HIV and others immunocompromised patients. The risk of HBV reactivation depends on host factors (including immunosuppression) and viral factors. It is now well established that immunocompromised patients can be classified into three groups for risk according to the type of immunosuppression and/or treatment. In patients at high risk, HBV therapy has to be considered systematically. In patients at moderate risk, the decision is based on the level of HBV DNA (preemptive treatment or monitoring and vaccination). In patients with low risk, HBV vaccination is another possible approach, although further studies are needed to assess the type of preemptive strategy.

## 1. Introduction

Hepatitis B Virus (HBV) is a pathogen that is prevalent worldwide and that can be transmitted either sexually, by blood, or from mother to child [1]. More than 2 billion people are infected by HBV worldwide [2]. In 2017, 257 million persons infected with HBV had chronic hepatitis B, which is associated with severe complications including cirrhosis and hepatocellular carcinoma, and with an estimated 887,000 deaths per year. Many individuals are considered cured subsequent to the development of hepatitis B surface antibodies (anti-HBs), which are the marker of protection against the disease.

Isolated anti-HBc (IAHBc) is a particular pattern of HBV serology, defined by negative hepatitis B antigens (Ag) and surface antibodies and positive anti-hepatitis B core antibodies (whether in IgM or IgG). The prevalence of this serological profile can be high, especially in patients infected with the hepatitis C virus (HCV), patients infected with human immunodeficiency virus (HIV), and other types of immunocompromised patients. Although this pattern can be a false-positive of the serology, it can also be found in patients with occult HBV infection. It is particularly important to screen immunocompromised patients for IAHBc since HBV replication can be reactivated with a potential for severe morbidity and mortality.

Although clinical guidelines have existed for some time, there is still a lot of debate regarding the management of the patients with IAHBc who are at risk of hepatitis B reactivation. Mostly, a prophylactic treatment is recommended for patients with the highest risk of reinfection/reactivation, and biological monitoring for other patients. Vaccination has been suggested in some cases, but there is no consensus, even though over the past decade, many researchers have studied the potential interest of vaccination in patients with IAHBc, especially in patients with HIV infection and concomitant HIV/HCV infection.

Herein, we summarize the current guidelines and new approaches to management in patients with IAHBc. We focus particularly on two types of patients: patients on immunosuppressive drugs for chronic inflammatory diseases (e.g., rheumatoid arthritis or Crohn disease) and HIV and/or HCV-infected patients. 

## 2. Pathophysiology and Phases of HBV Infection

### 2.1. Pathophysiology of HBV Infection

Hepatitis B virus is a *Hepadnaviridae*. The complete infecting virion (also called the Dane particle) is constituted of a capsid with a surrounding envelope. The virus contains the viral nucleic acid (DNA) and two enzymes (DNA-polymerase and kinase protein). The HBV genome is an open circular DNA molecule and there are four open-reading frames called P, S, C and X that code for viral proteins. 

**Figure 1:**
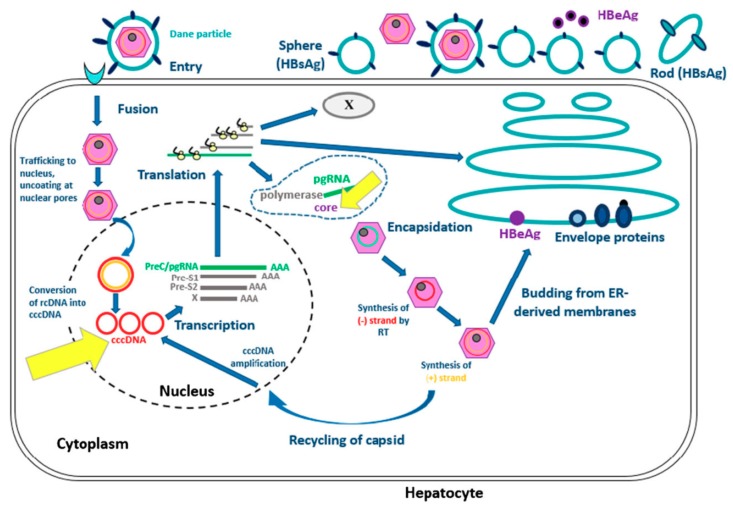
Hepatitis B virus life in hepatocyte [3,4]. Modified from Ait-Goughoulte M, Lucifora J, Zoulim F, Durantel D. Innate antiviral immune responses to hepatitis B virus. Viruses. 2010;2(7):1394-410.

The replicative cycle of HBV is currently well understood (See Figure 1, [3,4]). Attachment and entry of HBV involve interactions between HBsAg and surface proteins of the hepatocyte. Relaxed-circular DNA (rcDNA) is changed into covalently closed circular DNA (cccDNA) which is needed for transcription and production of new RNA and DNA, then matures thanks to the viral polymerase. The genome is used either for production of new virions and viral propagation outside the hepatocytes or for the constitution of a DNA reservoir in the nucleus of the host cell.

This figure represents the viral cycle of HBV from the entrance of the virus in the hepatocyte to the synthesis of new virions.

HBV DNA can persist in the hepatocyte in the form of cccDNA, unlike rcDNA which is the form found in the virus [5]. This viral genome is thus a reservoir [6] which persists even on nucleos(t)ide analogues, explaining why a cure for hepatitis B remains elusive [7]. The cccDNA also contributes to the synthesis of core antigen (HBcAg) and to the production of anti-HBc antibodies, explaining their presence in acute, chronic and resolved HBV infections.

HBV has little pathological effect on its own; it is driven mostly by the host’s immune response. However, this low pathogenicity is a traditional concept which is currently discussed. Indeed, effective control of HBV replication results in rapid ALT normalization. It indicates possible direct pathogenic role of HBV infection.

Four types of responses have been described. First, there can be a powerful immune response leading to complete and fast elimination of HBV and infected cells, resulting in acute hepatitis and even massive hepatocellular necrosis. Second, the immune response can be appropriate but weak, allowing what is often an asymptomatic infection to resolve progressively. Thirdly, the immune response may be weak and inadequate, with a state of partial tolerance resulting in considerable and prolonged HBV replication. Lastly, the immune response may be inexistent, which is often associated with asymptomatic carriage of HBV. 

Immune response involves innate, humoral and cellular immune systems. The innate response has an important role in hepatitis B physiopathology, since it is the first necessary step to trigger immune response. This involves both immune cells and inflammatory mediators, like for others infections. The humoral response is probably the most important because, after presentation of viral antigens (HBsAg), the B-cells will produce the neutralizing antibodies (anti-HBs) that confer effective immunity against the virus. Activated B-cells also activate T-cells, triggering a cytotoxic response that leads to the destruction of infected hepatocytes. 

### 2.2. Phases of Chronic HBV Infection

In 2017, the European Association for the Study of the Liver (EASL) proposed a new classification for chronic HBV infection. The classification contains four main stages, which are not necessarily sequential, and an additional fifth stage apart for occult HBV infection (OBI) (Table 1) [8]. The first stage refers to chronic infection with positive hepatitis B envelop antigens (HBeAg), high levels of HBV DNA and normal aspartate-aminotransferase (AST) and alanine-aminotransferase (ALT) levels. The second stage is chronic hepatitis with positive HBeAg, low level of HBV DNA and fluctuating ALT and AST levels. The third stage refers to chronic infection with negative HBeAg, weak HBV replication and normal ALT and AST levels (inactive carriers). The fourth stage is chronic hepatitis with negative HBeAg and fluctuating HBV DNA, AST and ALT levels.

## 3. Isolated anti-HBc Serological Patterns and Occult Hepatitis B Infection

### Definitions of Isolated Anti-HBc Pattern and Occult Hepatitis B Infection

The IAHBc serological pattern is defined as negative HBsAg and anti-HBs with positive anti-HBc. However, this pattern can vary [4]. First, it may be a false positive for anti-HBc, especially in low-prevalence populations. This false positivity has been estimated at between less than 10% to more than 50% [9,10,11,12,13,14,15]. Since this variation is associated with the biological assays used, the last generation of enzyme immunoassays must be used to reduce the risk of false positive. In general, it is recommended to re-test for the isolated anti-HBc pattern with another biological assay.

If the possibility of a false positive has been ruled out, this serological pattern may be a transient state of resolved HBV infection before the appearance of anti-HBs (in particular in acute HBV infection with immune complexes resulting from HBsAg—anti-HBs precipitation). However, the opposite may also be true, that is to say, a loss of anti-HBs because of waning immunity or the effect of treatments such as immunosuppressive drugs [16,17,18,19,20,21,22]. Another potential situation, chronic infection with a mutated HBsAg, is not recognized by standard serological assays.

The definition of OBI is not limited to an isolated anti-HBc pattern. OBI can be the association of negative HBsAg with positive HBV DNA in the blood and/or the liver [23], or positive HBV DNA in the liver whatever the level in the blood [24]. However, in European and American guidelines [8,25,26], the criterion of positive blood HBV DNA was retained to define OBI.

**Figure 2:**
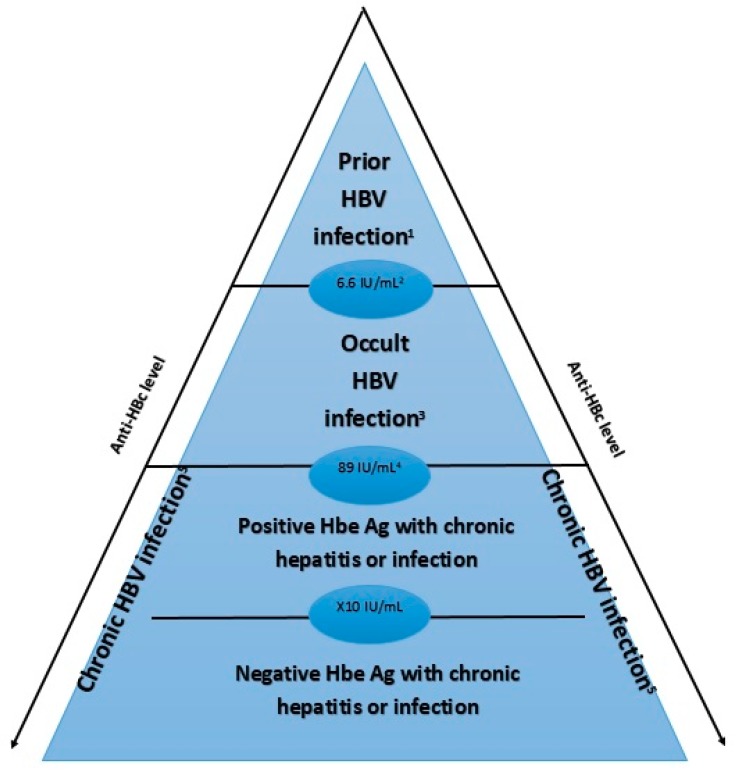
Level of anti-HBc depending on the stage of HBV infection. This figure represents the level of anti-Hbc according to the phase of HBV infection. It has been shown that anti-HBc level is higher in case of chronic HBV infection (especially in case of negative HbeAg) than in occult HBV infection and prior HBV infection. ^1^ Prior HBV infection: HBsAg -, anti-HBc +, HBV DNA -, ALT/AST normal ^2^ Cutoff 6.6 IU/mL: sensitivity of 60.7% and specificity of 75.3% ^3^ Occult HBV infection: HBsAg -, anti-HBc +, HBV DNA + ^4^ Cutoff 89 IU/mL: sensitivity of 95.8% and specificity of 98% ^5^ Chronic HBV infection: HBsAg + for ≥6 months *HBV: Hepatitis B Virus anti-HBc: Hepatitis B core antibody; HBeAg: hepatitis B e-antigen*.

The titration of anti-HBc may help distinguish OBI from other patterns of positive anti-HBc. Indeed, anti-HBc is produced by the immune system against HBcAg, a viral nucleocapsid protein which is the most immunogenic component of HBV. These antibodies are known as the marker of a prior HBV infection or exposition, and they can persist 10–20 years or more after viral clearance. The level of these antibodies is known to fluctuate depending on the stage of HBV infection [27]. It has been shown that the levels were higher in cases of chronic HBV hepatitis than in OBI, and higher in OBI than in cases of past/cured HBV infection [28]. In patients with an isolated anti-HBc profile, a cut-off of 6.6 IU/mL was associated with a sensitivity of 60.7% and a specificity of 75.3% for distinguishing OBI and previous infection. Finally, it has been shown that anti-HBc levels were higher in some stages of infections (Figure 2). Thus, a quantitative assessment of anti-HBc might be a straightforward means of distinguishing the different stages of HBV infection.

## 4. Epidemiology

In areas with low HBV prevalence, such as Europe and the United States, it has been estimated that IAHBc is found in between one and four and 10% of the population [15]. In one Korean study, the estimated prevalence in the general population was 8.9% (from 17,677 serum samples) [23]. The prevalence was higher in males and increased with age (0.7% in the ≤ 20 years age group; 1.9% in the 21–40 years age group; 7.4% in the 41–60 years age group; 17.1% in the 61–80 years age group; 24.2% in the >80 years age group) [23]. IAHBc also tends to be more frequent in areas with high prevalence of HBV infection, as observed in China which was found to have an overall prevalence of 11.9% [9].

This prevalence rises in some specific populations. For instance, in a prospective cohort of patients with IAHBc, 14.3% had an HIV coinfection and 40.5% had an HCV coinfection [29]. 

In persons living with HIV (PLHIV), the prevalence of IAHBc ranges from 7% to 40% [4,16,30,31,32,33,34,35,36,37,38,39,40,41,42,43,44] and seems associated with the defect in T-cell response. This prevalence was found to be higher in cases of intravenous drug use or alcohol abuse [30], with older age, low CD4 T-cell count (below 200 cells per µL) and detectable HIV RNA [45]. In studies of PLHIV with IAHBc, the prevalence of OBI can vary from 0% to 35% [31,36,46,47,48]. These variations may be explained by the different types of assays or the increasing use of dual-effect antiretroviral drugs including lamivudine, emtricitabine and tenofovir. These factors may lead to an underestimation of potential OBI, even though several cases of HBV reactivation in PLHIV with IAHBc have been described in the literature [36,37,49,50,51,52]. Occasionally, such reactivations have been reported following the withdrawal of antiviral drugs with dual anti-HIV/ant-HBV activity [53,54,55].

In patients on immunosuppressive drugs or chemotherapy, the prevalence of IAHBc ranges from 11% [56] to 18.1% [11].

In people infected with HCV, the prevalence of IAHBc is as high as 37% [30]. In this context, high prevalence is not explained by immunodepression but rather by the inhibitory effect of HCV on HBV replication, as suggested by lower viral loads and HBeAg levels in HBV/HCV coinfected patients than in patients with a single infection [57,58]. This mechanism has been explored in mouse models [59] which confirmed the likelihood that IAHBc prevalence is linked to the capacity of HCV core protein to inhibit HBV replication.

## 5. The Risk of HBV Reactivation Associated with IAHBc Pattern

The risk of HBV reactivation depends on host and viral factors [60]. Host factors include male sex, older age, presence of cirrhosis and type of immunosuppression. For example, the risk appears to be higher in patients with lymphoma than in rheumatologic diseases, like rheumatoid arthritis. HBV reactivation in PLHIV, which was initially more often linked to the progression of immunodepression, may be now linked to withdrawal of dual-action drugs for HIV and HBV.

HBV viral factors associated with a higher risk of reactivation are high baseline HBV DNA, positive HBeAg and associated HCV or HDV chronic hepatitis. Some studies have also suggested that non-A genotype HBV may be more prone to reactivation [61,62]. Hepatitis B reactivation is characterized by an abrupt increase in viral load and liver enzymes that can sometimes lead to liver failure and death [63].

The risk of HBV reactivation also depends on the type of immunosuppression and immunosuppressive treatment. In patients taking anti-CD20 antibodies treatments such as rituximab, B-cell immune response is impaired, with a decreased production of neutralizing antibodies (anti-HBs) [60]. The T-cell response is also impaired because B lymphocytes fail to present antigens to T lymphocytes. There is thus a high risk of HBV reactivation (more than 10%), especially in patients with malignant hematological diseases, and to a lesser degree, in those with rheumatoid arthritis [64,65,66,67,68]. In patients with malignant hematological diseases, one meta-analysis found a risk of HBV reactivation as high as 16.4% in patients on rituximab [69], even though the difference between the analyzed studies may have resulted from different definitions of HBV reactivation.

Although the mechanism is less clear, tumor necrosis factor α (TNFα) inhibitors also appear to be associated with potential HBV reactivation (from 0% to 8.3%) [66,70,71,72,73,74,75]. Recently, it was suggested that TNFα (but also IFN α/γ) can activate a unique host viral pathway, the APOBEC proteins that causes the degradation of cccDNA [76]. Thus, TNFα inhibitors can be associated with a risk of HBV reactivation and/or increased HBV replication. Patient on infliximab appears to be at a higher risk of HBV reactivation than those on etanercept, likely because infliximab is a more potent anti-TNF agent [65]. 

HBV reactivation with its potential consequences is particularly a concern when these people are exposed to either cancer chemotherapy, immunosuppressive or biologic therapies for the management of rheumatologic conditions, malignancies, inflammatory bowel disease, dermatologic conditions, or solid-organ or bone marrow transplantation [60].

The risk of reactivation has also been studied with newer biological molecules [66,77]. Abatacept is a fusion protein inhibiting CTLA4, which is an important mediator of lymphocyte communication and activation. However, although there are isolated reports of HBV reactivation [78], the risk seems very low for this therapy [79,80]. The situation is similar for the interleukin-6 inhibitor, tocilizumab, [81,82]. 

For corticosteroids, the exact mechanism of HBV reactivation remains unclear, but it is known that these molecules have a role in the activation of HBV replication [66]. The use of glucocorticoids has been associated with an increased risk of HBV reactivation [83,84,85]. However, they are often taken with another immunosuppressive drug, making it difficult to distinguish the role of glucocorticoids alone. Nevertheless, the risk seems associated with the cumulative dose of corticosteroids, and is categorized as either high (more than 20 mg/day for at least 4 weeks), moderate (10–20 mg/day for at least 4 weeks) or low (less than 10 mg/day for less than 4 weeks). 

Traditionally, immunosuppressive drugs (such as methotrexate, azathioprine, sulfasalazine, or leflunomide) have also been described as possibly responsible for HBV reactivation, though only a few cases have been reported [86,87,88,89]. These treatments are therefore classified in the low-risk group. For methotrexate, the risk of HBV reactivation seems paradoxically to occur after the discontinuation of treatment: restored immune response prompts an increase in the destruction of hepatocytes, resulting in HBV reactivation through the release of virions [66]. 

The mechanism of HBV reactivation during HCV infection is related to HCV therapy, which lift the inhibition of HCV on HBV replication [90]. This was observed more often with direct acting antiviral agents than with interferons alpha-based treatments [90], possibly because of the dual anti-HCV/anti-HBV activity of interferons-alpha. Overall, the risk is very low [90,91,92]. However, reactivation of HBV may arise in patients without control of HBV replication by nucleos(t)id analogues, with potentially severe liver damage [93].

## 6. The Management of Patients with the IAHBc Pattern Who are Immunosuppressed or at Risk of Immunosuppression

It should be highlighted that each patient who is immunosuppressed or at risk of immunosuppression, has to be tested for HBV serology and in cases of prolonged immunosuppression, testing has to be repeated if negative initially.

When faced with an IAHBc serological pattern, it is first recommended to rule out a false positive with a second assay. An additional assay has to be done between 1 and 3 months after the first serology to assess whether anti-HBs has appeared. If not, blood HBV DNA quantification should be used to investigate the presence of OBI. 

To a lesser degree and as previously reported [28], the quantification of anti-HBc antibodies may be of help in distinguishing prior HBV infection from occult or ongoing HBV infection (Figure 2). 

The risk of hepatitis B reactivation has to be assessed taking into account the associated comorbidities, the type of immunosuppression and/or the immunosuppressive treatment taken by the patient. According to these characteristics, risk may be classified into three categories: high (10% and more), moderate (1% to 10%) or low (less than 1%) (Table 2). Because no study has confirmed whether the quantification of anti-HBc antibodies could be used better determine the risk of reactivation, this criterion cannot be included in the current recommendations. 

**Figure 3:**
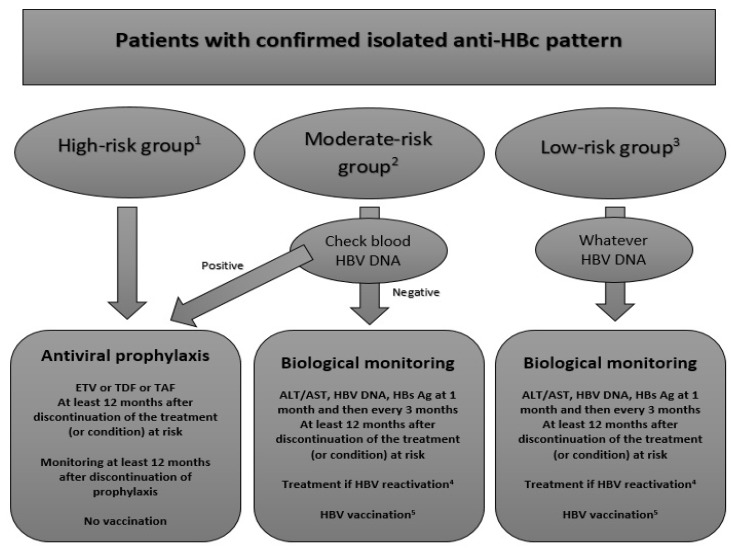
Proposed algorithm for the management of patients with IAHBc and immunosuppressive treatment or condition. The figure represents our proposed algorithm to manage immunocompromised patients with IAHBc according to three different risk groups. ^1^ High-risk group: B-cell depleting agents as rituximab ^2^ Moderate-risk group: HIV infection, TNFα inhibitors, corticosteroids at high or moderate doses, systemic cancer chemotherapy, cytokine-based therapies, immunophilin inhibitors, tyrosine kinase inhibitors, proteasome inhibitors, histone deacetylate inhibitors ^3^ Low-risk group: corticosteroids at low dose or intra-articular, Abatacept, Tocilizumab, Methotrexate, Azathioprine, 6-mercaptopurine, Direct antiviral agents for HCV ^4^ HBV reactivation: seroconversion of HBsAg and detectability of HBV DNA ^5^ HBV vaccination: as in the general population with 3 doses of vaccine at 20µg with a control of anti-HBs one month after the last dose *ETV: entecavir; TDF: tenofovir diproxil fumarate; TAF: tenofovir alafenamide; ALT: alanine-aminotransferase; AST: aspartate-aminotransferase*.

However, patients could be managed according to this classification following the algorithm presented in Figure 3.

In the high-risk group, the current guidelines recommend systematically the prescription of a preemptive treatment by entecavir. 

According to most recommendations, we think that a preemptive treatment for HBV reactivation has to be prescribed no matter the blood HBV DNA level [8,25,26]. Lamivudine has to be avoided considering its low genetic barrier [26], and entecavir was only found to be superior in one study in hematological patients on rituximab [94]. Prospective data on the use of tenofovir are still lacking, ant it can be speculated that the new formulation, tenofovir alafenamide (TAF), will limit the risk of renal toxicity for long-term treatment. 

Preemptive therapy with entecavir or tenofovir has to be continued at least 12 months after the discontinuation of immunosuppressive treatment seeing as the risk of HBV reactivation could persist after the discontinuation of treatments such as CD20 inhibitors. While on preemptive treatment, patients should be tested for ALT, AST, HBsAg and HBV DNA every 3 months. 

The benefit and interest of HBV vaccination has not yet been assessed and cannot be widely recommended. Furthermore, considering the blockade of antibody production by B-cell depleting agents, only reinforced short-course vaccinations schemes (which have not yet been studied) could be used in these patients, since the introduction of these agents most often cannot be delayed for months. Thus, HBV vaccination of patients with IAHBc could be another potential management option but mainly in HIV-infected patients and for those on TNFα inhibitors. 

In the moderate-risk group (mainly PLHIV and/or on TNFα inhibitors), the current guidelines recommend to sample the blood HBV DNA level. If detectable, a preemptive treatment will be prescribed whereas if it is undetectable, simple monitoring will be required.

With the recent data, we also think that patient management is mainly driven by the HBV DNA level in the blood. In patients with detectable HBV DNA (i.e., OBI), a preemptive treatment has to be introduced with the same rules about duration and monitoring as in the high-risk group. HBV vaccination could be of interest in patients with detectable HBV DNA but has not been clearly defined yet. 

Simple monitoring is considered appropriate for patients with undetectable HBV DNA. HBsAg, ALT, AST and HBV DNA have to be monitored one month after the initiation of treatment (if any) and then every 3 months afterwards. If HBV reactivation is detected, patients should be started on an appropriate course of treatment. However, in patients without detectable HBV DNA in the blood and particularly in PLHIV, vaccination seems to be of particular interest. Although randomized controlled trials are lacking, six prospective cohorts or trials using different schemes have assessed the vaccine response in this population [95,96,97,98,99,100] (Table 3). All these studies suggested that vaccination could be used to prevent HBV reactivation. However, the rate of anamnestic response varied dramatically between studies. The best way to manage patients with an anamnestic response to a single dose has to be defined, although it can be speculated that they could followed the same regime as low-risk patients. Furthermore, even though the reinforced scheme (three double doses) seems to be the best (as also observed in PLHIV without HBV serological markers or in those who failed to respond to standard vaccination), it has not been evaluated in other populations of interest. 

Some vaccination studies have been conducted in patients on TNFα and immunomodulatory therapies [101,102,103,104,105,106,107,108]. These studies reported rates of patients with anti-HBs antibodies > 10 UI/L after vaccination ranging between 16.7% and more than 80%, and between 12.5% and 40% of patients with anti-HBs > 100 UI/L. These variations may be explained, similarly to the studies in PLHIV, by the different vaccination schemes used in the studies. The vaccine response was lower in patients on TNFα inhibitors, especially infliximab. 

HBV vaccination (standard or reinforced scheme) is an option worth considering for patients with IAHBc without detectable HBV DNA who are at moderate risk of HBV reactivation. 

In the low-risk group, only monitoring is currently required by all the guidelines. Indeed, HBV DNA has no effect on the management of the IAHBc patient, but it should be investigated regardless. Preemptive treatment is not recommended, even in patients with detectable blood HBV DNA. Although the guidelines recommended monitoring low-risk patients similarly to moderate-risk patients, it seems fitting to suggest vaccination even though reactivation is unlikely. This situation (patients at low-risk) is likely similar for people with IAHBc without present or predicted future immunodepression. We can assume that everybody has a potential risk of immunodepression in the future, and therefore, the sooner preventive measures are taken, the better. 

## 7. Conclusions

The clinical importance of IAHBc pattern had risen from relative obscurity as a result of the growing rate of patients on immunosuppressive drugs or with an immunosuppression. This type of pattern can be a challenge for clinicians since it potentially reflects different physiopathological situations, from false positive to genuine occult hepatitis B. In addition, while not rare in the general population, the prevalence of the IAHBc pattern appears to be higher in at-risk populations who are influenced by a number of complex mechanisms, underlining the need for cautious management. It is commonly stated that preemptive HBV therapy should be provided to IAHBc patients at higher risk of HBV reactivation, but the potential effect and the approach to HBV vaccination need to be further studied. New studies focusing specifically on people harboring the serological pattern of interest are needed to enhance our global knowledge at an individual level and to refine the current general recommendations. 

## Figures and Tables

**Table 1:** New classification of the phases of HBV infection.

	*Positive HBeAg* *Chronic Infection Chronic Hepatitis*		*Negative HBeAg* *Chronic Infection Chronic Hepatitis*		*Occult HBV Infection*
***HBsAg***	**High**	High/Intermediate	Low	Intermediate	Negative
***HBV DNA*^1^**	≥10^7^ IU/mL	10^4^–10^7^ IU/mL	<2000 IU/mL	>2000 IU/mL	Positive
***ALT/AST***	Normal	Elevated	Normal	Elevated	Normal
***Liver disease***	None/Minimal	Moderate/Severe	None	Moderate/Severe	Variable
***Old terminology***	Immune tolerant	Immune reactive HBeAg positive	Inactive carrier	HBeAg negative chronic hepatitis	/

^1^ HBV DNA in blood HBsAg: Hepatitis B surface Antigen; HBeAg: soluble antigen “e”; HBV DNA: Hepatitis B desoxyribonucleic acide; ALT: alanine-aminotransferase; AST: aspartate-aminotransferase; IU: International Units.

**Table 2:** Classification of IAHBc patients according to the risk of HBV reactivation.

High Risk (≥10%)	Moderate Risk (1%–10%)	Low Risk (<1%)
	HIV infection	
B-cell depleting agents (rituximab)	Corticosteroids at high ^1^ or moderate doses ^2^ TNFα inhibitors (infliximab > others) Systemic cancer chemotherapy (Anthracycline derivatives> others) Cytokine-based therapies Immunophilin inhibitors Tyrosine kinase inhibitors Proteasome inhibitors Histone deacetylate inhibitors	Low dose ^3^ or intra-articular corticosteroids Abatacept Tocilizumab Methotrexate Azathioprine, 6-mercaptopurine DAAs

^1^ High dose: at least 20 mg per day for at least 4 weeks ^2^ Moderate dose: between 10 and 20 mg per day for at 4 weeks ^3^ Low dose: less than 10 mg per day or less than 4 weeks *IAHBc: Isolated anti-HBc; TNF: Tumor Necrosis Factor; HIV: Human Immunodeficiency Virus; DAAs: Direct Antiviral Agents*.

**Table 3:** HBV Vaccination in HIV-infected patients with isolated anti-HBc.

Study (year)	Design	Schedule	Anamnestic Response ^1^	Total Response Rate	Predictor Factors
**Morsica et al. (2017)** **[92]**	Prospective *n* = 25	20 µg at W0 3 doses if anti-HBs < 100 IU/mL after first dose	24%	52.6%	Presence of OBI HCV co-infection
**Piroth et al. (2016)** **[93]**	Prospective *n* = 54	20 µg at W0 then 40 µg at W5–9–24 (if non-AR)	46%	W28: 89% M18: 81%	NA
**Kaech et al. (2012)** **[94]**	Prospective *n* = 37	20 µg M0 +/− 20 µg M0-1-6	22%	60%	Injected drug use
**Chakvetadze et al.** **(2010)** **[95]**	Prospective *n* = 40	20 µg +/− 2-5 doses 20 µg	32.5%	40.7% W10 64% W21	None
**Jongjirawisan et al. (2006)** **[96]**	Prospective *n* = 140	20 µg once	7%	NA	Intravenous drug use, HCV co-infection
**Gandhi et al. (2005)** **[97]**	Prospective *n* = 69	20 µg W0-4-24	16%	62%	Male, HCV co-infection

^1^ AR: Anamnestic response: anti-HBs > 10 IU/mL after one dose of vaccine µg: micrograms; OBI: occult hepatitis B infection; HCV: hepatitis C virus; W: week; NA; not assessed; M: month.
